# Seven-Signal Proteomic Signature for Detection of Operable Pancreatic Ductal Adenocarcinoma and Their Discrimination from Autoimmune Pancreatitis

**DOI:** 10.1155/2012/510397

**Published:** 2012-05-14

**Authors:** Kiyoshi Yanagisawa, Shuta Tomida, Keitaro Matsuo, Chinatsu Arima, Miyoko Kusumegi, Yukihiro Yokoyama, Shigeru B. H. Ko, Nobumasa Mizuno, Takeo Kawahara, Yoko Kuroyanagi, Toshiyuki Takeuchi, Hidemi Goto, Kenji Yamao, Masato Nagino, Kazuo Tajima, Takashi Takahashi

**Affiliations:** ^1^Institute for Advanced Research, Nagoya University, Furo-cho, Chikusa-ku, Nagoya 464-8601, Japan; ^2^Division of Molecular Carcinogenesis, Center for Neurological Diseases and Cancer, Nagoya University Graduate School of Medicine, Nagoya 466-8550, Japan; ^3^Division of Epidemiology and Prevention, Aichi Cancer Center, Nagoya 464-8681, Japan; ^4^Division of Research and Development, Oncomics Co., Ltd, Nagoya 464-0858, Japan; ^5^Division of Surgical Oncology, Department of Surgery, Nagoya University Hospital, Nagoya 466-8550, Japan; ^6^Department of Gastroenterology, Nagoya University Hospital, Nagoya 466-8550, Japan; ^7^Department of Gastroenterology, Aichi Cancer Center, Nagoya 464-8681, Japan

## Abstract

There is urgent need for biomarkers that provide early detection of pancreatic ductal adenocarcinoma (PDAC) as well as discrimination of autoimmune pancreatitis, as current clinical approaches are not suitably accurate for precise diagnosis. We used mass spectrometry to analyze protein profiles of more than 300 plasma specimens obtained from PDAC, noncancerous pancreatic diseases including autoimmune pancreatitis patients and healthy subjects. We obtained 1063 proteomic signals from 160 plasma samples in the training cohort. A proteomic signature consisting of 7 mass spectrometry signals was used for construction of a proteomic model for detection of PDAC patients. Using the test cohort, we confirmed that this proteomic model had discrimination power equal to that observed with the training cohort. The overall sensitivity and specificity for detection of cancer patients were 82.6% and 90.9%, respectively. Notably, 62.5% of the stage I and II cases were detected by our proteomic model. We also found that 100% of autoimmune pancreatitis patients were correctly assigned as noncancerous individuals. In the present paper, we developed a proteomic model that was shown able to detect early-stage PDAC patients. In addition, our model appeared capable of discriminating patients with autoimmune pancreatitis from those with PDAC.

## 1. Introduction

Pancreatic ductal adenocarcinoma (PDAC) is the fifth leading cause of cancer death in Japan with more than 24,000 deaths annually [1], while 35,000 deaths each year in the United States are caused by the disease [2]. Long-term survival for PDAC patients remains unsatisfactory, with only 3–5% surviving for more than 5 years after surgical resection, with the remainder succumbing to widespread metastasis or massive local recurrence. Since surgical resection is the only reliable curative treatment, early detection is essential to improve the outcomes of affected individuals. However, the clinical symptoms of PDAC are often unremarkable until advanced stages of the disease, and the anatomic location of the pancreas deep in the abdomen makes physical detection and imaging approaches difficult. Thus, less than 10% of patients diagnosed with PDAC are eligible for surgical resection [3]. Although serum markers for PDAC including carcinoembryonic antigen (CEA) and carbohydrate antigen 19-9 (CA19-9) play important roles in current clinical practice for monitoring progression and treatment response, as well as surveillance for recurrence, these markers are not ideal for cancer screening due to their low specificity and/or sensitivity in early stages of the disease [4–6].

The concept of autoimmune pancreatitis (AIP) is supported by recent advances in elucidating its pathogenesis as a unique systemic disease. AIP has several characteristic features, such as infiltration of CD4-positive T cells and IgG4-positive plasmacytes, irregular narrowing of the pancreatic duct, and diffuse enlargement of the pancreas [7–9]. Although intensive investigations into the pathogenesis of AIP have been conducted, its underlying molecular mechanism remains unclear. The most important and difficult step in diagnosing AIP is to distinguish it from PDAC. Clinical symptoms such as obstructive jaundice are not helpful for discrimination, while IgG4, the most accurate serum marker for AIP, is not adequately specific to exclude the existence of cancer. Furthermore, AIP is sometimes accompanied by PDAC; thus percutaneous or endoscopic biopsy findings are often needed for final diagnosis. Unfortunately, those examinations are invasive for the patient and may fail to detect small regions of cancer cells. As a result, unnecessary surgery because of misdiagnosis performed for AIP patients without cancer or those undergoing treatment for existing cancer is a critical issue in clinical practice. Accordingly, there is urgent need for elucidation of novel biomarker(s) and noninvasive diagnostic strategies useful for early detection of PDAC, as well as discrimination of patients with AIP to improve clinical management and prognosis.

 Comprehensive analysis of protein expression patterns in biological materials might improve understanding of the molecular complexities of human diseases [10] and could be useful to detect diagnostic or predictive protein expression patterns that reflect clinical features. Matrix-assisted laser desorption/ionization mass spectrometry (MALDI MS) can profile proteins up to 50 kDa in size in serum, tissues, and other various clinical specimens. Protein profiles obtained may contain thousands of data points and provide proteomic signatures that allow detection of patients with various diseases [11, 12]. We previously employed MALDI MS for expression profiling of proteins in human lung cancer specimens and found that the resultant proteomic patterns could predict various clinical features, as well as the potential of recurrence in stage I lung cancer patients [13, 14].

In the present study, protein expression profiling with MALDI MS was conducted to identify proteomic patterns in plasma samples for discrimination of PDAC from AIP as well as chronic pancreatitis (CP) using 3 independent datasets. We found that a proteomic model consisting of 7 mass spectrometry signals constructed by use of the training cohort could detect 82.6% (38 of 46, 95% CI 68.6–92.2) of known PDAC cases, including 62.5% (5 of 8, 95% CI 24.5–91.5) of the stage I and II cases in the independent test cohort, which successfully confirmed its discrimination power. We further applied our model for discrimination of AIP as well as CP from PDAC and found that it correctly assigned 100% of the AIP and CP patients (19 of 19, 95% CI 82.4–100 and 11 of 11, 95% CI 71.5–100, resp.) as noncancerous. These results indicate that our 7-signal proteomic model may contribute to accurate decisions regarding the therapeutic plan for patients with chronic pancreatic diseases, especially PDAC and AIP.

## 2. Methods

### 2.1. Patients and Specimens

Plasma specimens from 96 PDAC patients were obtained from the Department of Epidemiology and Prevention, Aichi Cancer Center Research Institute, Nagoya, Japan, collected from January 2001 and November 2005. Of those, 80 were randomly assigned to the training set and 16 to the test set. An additional 30 plasma specimens from PDAC patients were obtained from the Department of Surgery, Nagoya University Hospital, Nagoya, Japan, collected from May 2004 to July 2006, and assigned to the test set. Plasma specimens from 147 healthy control subjects were also obtained from the Department of Epidemiology and Prevention, Aichi Cancer Center Research Institute, and used. Of those, 80 were randomly assigned to the training set and 67 to the test set. Plasma specimens from 2 acute pancreatitis, 11 chronic pancreatitis, and 3 autoimmune pancreatitis patients were obtained from the Department of Gastroenterology, Nagoya University Hospital, collected from April 2005 and November 2007, and assigned to the test set. In addition, 16 plasma specimens from autoimmune pancreatitis were obtained from the Department of Gastroenterology, Nagoya University Hospital, collected from September 2003 and August 2009, and assigned to the confirmation set. More detailed information is available in Supplementary Material available on line at doi: 10.1155/2012/510397. The characteristics of the patients and healthy subjects in the training, test, and confirmation cohorts are summarized in Supplementary Table S1, which shows that there were no statistically significant differences in regard to clinicopathologic features among the cohorts. All specimens were processed in the same manner and stored at −80°C within 180 minutes after being collected from the patients and healthy subjects, and not thawed until analysis. Requisite approval from our institutional review boards and written informed consent from all subjects were obtained. One plasma specimen per patient or healthy subject was analyzed, and the training, test, and confirmation datasets were independently analyzed as different batches. Further details are available in supplementary Material.

### 2.2. Proteomic Analysis

Five microliters of nonpre-treated plasma was mixed with 5 nL drops of an energy absorbing matrix solution (saturated Sinapinic acid in water/acetonitrile/trifluoroacetic acid (500 : 500 : 1, by volume), which allows molecules to be protonated and desorbed from tissue surfaces). Then, 1 *μ*L mixtures were deposited into individual wells of MALDI MS sample plates (PE Biosystems, Foster City, CA) and dried at room temperature for 5 minutes. Six spots were generated for each plasma-matrix mixture sample and spectra were acquired from all 6 using a 4800 Instrument (Applied Biosystems, Foster City, CA), essentially as described previously [13, 14]. Further details are available in Supplementary Material.

### 2.3. Statistical Methods

Protein profiles obtained by MALDI MS were analyzed using 3 distinct statistical methods, Fisher's exact test, the Kruskal-Wallis test, and a significance analysis of microarray (SAM) test [15], to investigate MS signals that appeared to differentiate PDAC patients from healthy individuals in the training set. MS signals that met at least 1 of the 3 selection criteria were further analyzed.

To construct a generally applicable proteomic classifier without specifically overfitting it to the training cohort, we used a weighted voting algorithm, a well-established technique for supervised classification, in which each weight value was calculated as the signal-to-noise ratio and a leave-one-out cross-validation strategy was utilized [16].

 It is possible that unintended biased resubstitution or partial cross-validation can result in underestimation of the error rate after cross-validation; thus the performance of any class prediction rule is best assessed by applying the rule created by use of 1 dataset (the training set) to an independent dataset (the validation or test set) [17]. In the present study, the proteomic classifier constructed with the training dataset of 160 individuals was validated using a completely independent validation set composed of 145 individuals.

An agglomerative hierarchical clustering algorithm was applied to investigate the pattern among the statistically significant discriminator proteins as well as the biological status with Eisen's software [18].

### 2.4. Identification of Individual Proteins in the Proteomic Signature

 40 *μ* of serum samples was pretreated with high abundant protein depletion column (Agilent, Palo Alto, CA) according to manufacturer's instruction. The pretreated serum samples were separated over a polymeric column (Toso, Tokyo, Japan) with a high-performance liquid chromatography (HPLC) pump (Shimadzu, Osaka, Japan) and HPLC fractions were collected every minute for 80 minutes. Each fraction was lyophilized, reconstituted with a 50% acetonitrile in water containing 0.1% trifluoroacetic acid, and analyzed by MALDI mass spectrometry to identify the HPLC fractions that contained proteins corresponding to the peaks in the signature with molecular weights selected by bioinfomatic analysis as candidate molecular markers for the PDAC. The selected fractions were lyophilized and reconstituted with a mixture of 10 *μ*L of 0.4 M ammonium hydrogen carbonate and 5 *μ*L of 45 mM dithiothreitol, and then 10 *μ*L of 100 mM iodoacetamide was added. This mixture was incubated for 4 hours at 37°C with 5 *μ*L of 200 nM mass-grade trypsin (Promega, Madison, WI) to obtain peptides. The peptides were separated and sequenced by a microcapillary reverse-phase column (KYA technologies, Tokyo, Japan) with an HPLC pump (KYA) and MALDI mass spectrometer (Applied Biosystems). These spectra were compared with those in the human databases of the National Center for Biotechnology Information (nonredundant) by use of Mascot version 2.1.0 (Matrix Science Inc., Boston, MA). A minimum of two peptide matches and a positive association between the m/z values detected with MALDI mass spectrometry and the molecular weight of the intact protein (including posttranslational modifications) were required for protein identification.

## 3. Results

### 3.1. Protein Expression Profiling in the Training Cohort

**Figure 1:**
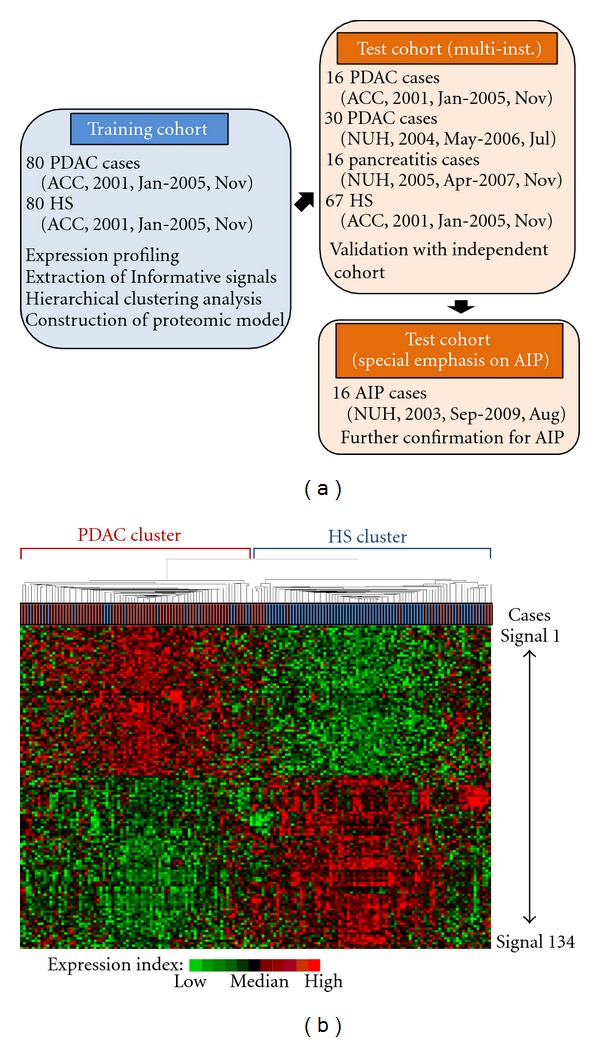
MALDI MS analysis of plasma specimens from human PDAC patients and healthy subjects in the training cohort. (a) Independent training-validation-confirmation datasets of 160 training cases, 129 validation cases, and 16 confirmation cases. (b) Unsupervised hierarchical clustering analysis of 80 human PDAC patients and 80 healthy subjects in the training cohort according to the protein expression patterns of 134 MS signals. Each row represents an individual proteomic signal and each column an individual sample. The dendrogram at the top shows the similarities in protein expression profiles among the samples. Substantially elevated (red) expression of the proteins was observed in individual plasma samples. HS: healthy subjects; PDAC: pancreatic ductal adenocarcinoma. Red box case: PDAC: blue box case: healthy subject.

**Figure 2:**
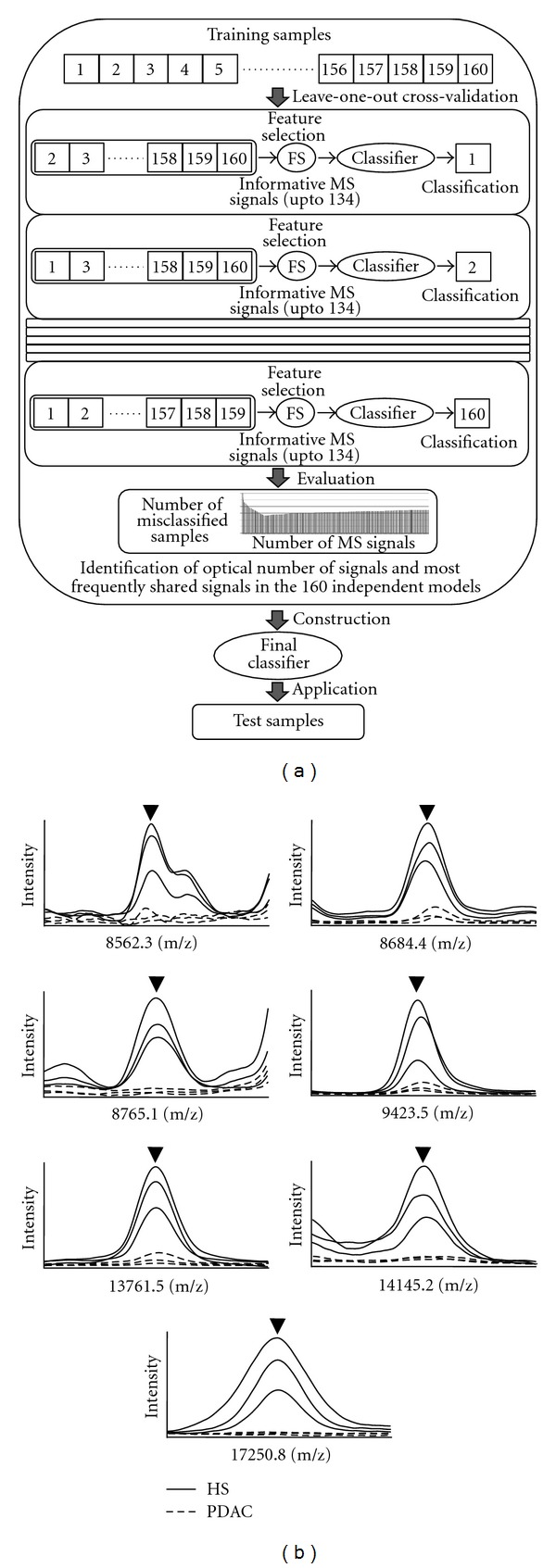
Construction of proteomic model for discrimination of PDAC cases from healthy subjects. (a) Schematic diagram of construction of proteomic discrimination model. (b) Representative mass spectra comprising 7-signal proteomic signature. Arrowheads show informative peaks for discrimination between healthy subjects and PDAC patients. Blue lines show representative spectra from healthy subjects and red lines show representative spectra from PDAC patients.

 We obtained protein expression profiles for the 160 human plasma specimens obtained from 80 PDAC patients and 80 healthy subjects at Aichi Cancer Center (Figure 1(a)) and Supplementary Table S1) using MALDI MS. Spectra were obtained from 6 replicates of single plasma specimens. MarkerView (Applied Biosystems) and custom software were used to bin the peaks across the spectra obtained from 960 samples, and then we calculate the average intensity of each signal individually among the 160 cases. As a result, we obtained expression profiles containing 1063 distinct proteomic signals. To extract a proteomic signature able to discriminate PDAC patients from healthy individuals, we compared MS signals from the 80 healthy subjects and 80 PDAC patients using our statistical selection criteria (signals met at least 2 of the following criteria: *P* value corrected with Bonferroni was less than 0.05 in Fisher's exact test and Kruskal-Wallis test, and FDR < 0.1% for SAM). As a result, 134 MS signals were found to be differentially expressed. Agglomerative hierarchical clustering analysis using the identified proteomic signature showed a clear separation of plasma specimens from PDAC patients as compared to those from healthy individuals (Figure 1(b)), which confirmed that the selected MS signals were informative for discrimination of PDAC cases from healthy individuals. The left branch mostly consisted of PDAC cases (81.3%, 65 of 80 cases, 95% CI 71.0–89.1), whereas the right branch consisted of healthy subjects (78.8%, 63 of 80 cases, 95% CI 68.2–87.1). Next, we investigated whether our proteomic prediction model could best distinguish noncancerous individuals from cancer patients. For this purpose, the 134 selected MS signals, which were informative for discrimination, were further ranked according to the SAM and weighted-voting proteomic discriminatory models were constructed using increasing numbers of the differentially expressed proteomic signals (up to 134), for which learning errors were calculated by leave-one-out cross-validation (Figure 2(a)). This cross-validation analysis showed that the use of 7 MS signals gave the lowest number of misclassifications, while 7 MS signals (8562.3, 8684.4, 8765.1, 9423.5, 13761.5, 14145.2, and 17250.8 m/z) were extracted as the most shared ones. Using this proteomic model, plasma samples from both PDAC patients and healthy subjects were classified as either positive or negative for cancer, which showed that the sensitivity for prediction was 76.3% (61 of 80 of the cancer patients, 95% CI 65.4–85.1) and for specificity was 91.3% (73 of 80 of the healthy subjects, 95% CI 82.8–96.4, Table 1), for an overall classification accuracy of 83.8% (134 of 160, 95% CI 77.1–89.1). We also calculated positive and negative predictive values (PPV and NPV, resp.) to confirm the diagnostic power of our model, which were 89.8% and 79.3%, respectively. We observed no significant difference for detection of PDAC patients related to lymph node positivity and prognosis. Furthermore, we analyzed the relationship between the age of PDAC patients (≤60 or >60 years old) and detection power of the 7 MS signals. Those results showed that the sensitivity for prediction was 69.8% (30 of 43, 95% CI 53.9–82.8) and 83.8% (31 of 37, 95% CI 68.0–93.8) in the younger and older groups, respectively (Table 1), with no significance in discrimination found (*P* = 0.142, Fisher's exact test). Representative spectra that comprised the 7-signal proteomic model for the healthy subjects and PDAC patients are shown in Figure 2(b). It is of note that our model was able to correctly distinguish 72.7% (8 of 11 cases, 95% CI 39.0–94.0) of the stage I and II cases from the healthy subjects, while it also correctly classified 78.8% (26 of 33, 95% CI 61.1–91.0) of the PDAC patients eligible for surgical resection as positive for cancer (Table 1).

### 3.2. Protein Expression Profiling in the Test Cohort

**Figure 3:**
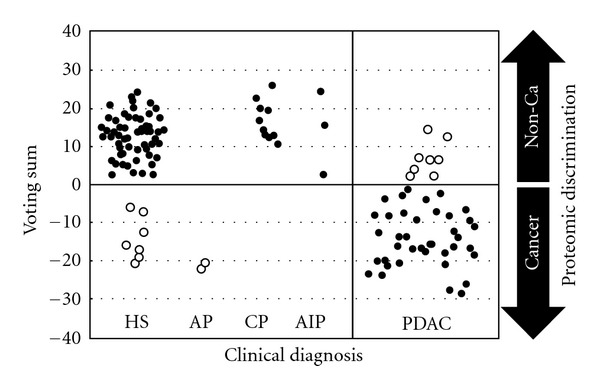
Assessment of 7-signal proteomic model with the validation cohort using weighted voting algorithm. The results of proteomic analyses of the training cohort are shown. Each circle represents a voting sum for a single patient. Solid circles: specimens whose prediction with proteomic model matched clinical diagnosis; open circles: specimens whose prediction with proteomic model did not match clinical diagnosis; HS: healthy subjects; AP: acute pancreatitis; CP: chronic pancreatitis; AIP: autoimmune pancreatitis; PDAC: pancreatic ductal adenocarcinoma.

It has been well reported that the robustness, including accuracy, of a prediction model should be assessed using an independent validation cohort, even when cross-validation methods, such as LOOCV or n-fold CV, were properly used for developing the prediction model [19]. To examine the robustness of the 7-signal proteomic model constructed with data from MALDI-MS analysis of the training cohort, we applied it to an independent test dataset obtained from plasma samples collected at two different institutions. We also determined whether the identified proteomic model could discriminate between acute and chronic pancreatitis patients, as well as autoimmune pancreatitis, as the discovery of biomarkers applicable for differential diagnosis between PDAC and noncancerous pancreatic diseases has great potential for clinical practice. For the test cohort, plasma samples were obtained from 46 PDAC patients (16 and 30 cases of ACC and NUH, resp.) and 67 healthy subjects from the ACC group, while 16 pancreatitis samples obtained from Nagoya University hospital (NUH) consisted of 2 acute pancreatitis, 11 chronic pancreatitis, and 3 autoimmune pancreatitis cases (Figure 1(a), Supplementary Tables S1 and S2 for additional clinical information for AIP patients). With the 7-signal proteomic model, 82.6% (38 of 46, 95% CI 68.6–92.2) of the cancer cases were classified into the positive group, while 89.2% (74 of 83, 95% CI 80.4–94.9) of the noncancerous subjects were assigned to the group negative for cancer (Figure 3 and Table 2). We calculated PPV and NPV, which were 80.9% and 90.2%, respectively, and the overall accuracy of the classification with the test cohort was 86.8% (112 of 129, 95% CI 79.7–92.1). We also evaluated the relationship between blood vessel invasion (surgery with or without mesenteric venous tract resection) and detection power of the 7 MS signals. Our results showed that the sensitivity for prediction was 88.8% (8 of 9, 95% CI 51.8–99.7) for PDAC patients who underwent mesenteric venous tract resection and 78.6% (11 of 14, 95% CI 49.2–95.3) for those who did not, with no significant difference found (*P* = 0.524, Fisher's exact test). Future studies with a larger number of PDAC patients treated with surgery are warranted to validate the clinical usefulness of our 7-signal proteomic signature. It is of note that our model was able to correctly distinguish 62.5% (5 of 8 cases, 95% CI 24.5–91.5) of the stage I and II cases from the healthy subjects and also classified 78.9% (30 of 38, 95% CI 62.7–90.5) of the PDAC patients eligible for surgical resection as positive for cancer. It is also noteworthy that the identified proteomic model distinguished 100% of the patients with chronic pancreatitis (11 of 11, 95% CI 71.5–100) and AIP (3 of 3, 95% CI 29.2−100) from cancer cases (Figure 3 and Table 2).

### 3.3. Discrimination of Autoimmune Pancreatitis from PDAC Using 7-Signal Proteomic Model

**Figure 4:**
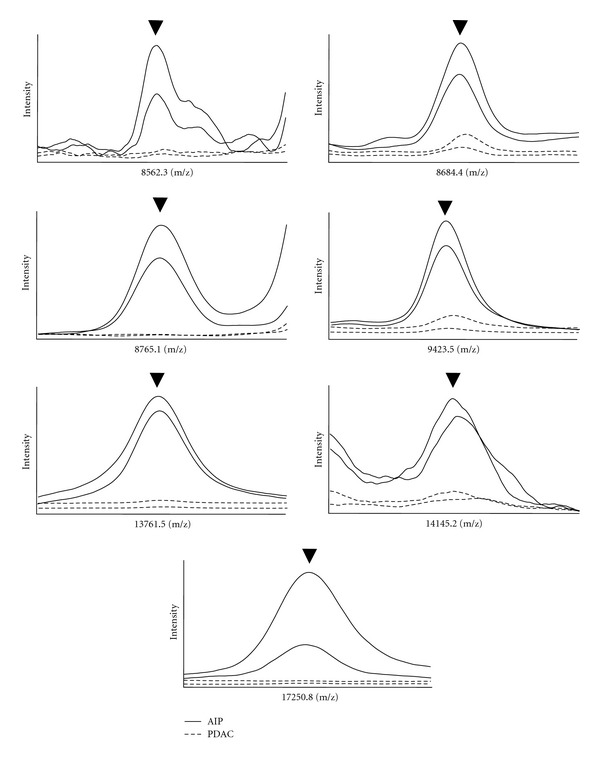
Representative mass spectra comprising 7-signal proteomic signature in autoimmune pancreatitis patients and PDAC patients. Arrowheads show informative peaks for discrimination between autoimmune pancreatitis patients and patients with pancreatic cancer. Blue solid and dotted lines show representative spectra from autoimmune pancreatitis patients, and red solid and dotted lines show representative spectra from pancreatic cancer patients. AIP: autoimmune pancreatitis; PDAC: pancreatic ductal adenocarcinoma.

Autoimmune pancreatitis is a systemic inflammatory disease of the pancreas and several diagnostic criteria have been proposed. However, their usefulness is under debate and accurate differential diagnosis remains difficult. Moreover, an important step in diagnosing AIP is to discriminate it from PDAC. In the present study, all (3 of 3) of the AIP patients were correctly discriminated from those with PDAC in the analysis with the test dataset; thus we next performed a confirmatory analysis using plasma samples collected from 16 AIP patients treated at NUH (Figure 1(a) and Supplementary Table S2). For this, we employed our 7-signal proteomic model to investigate whether it would classify the AIP patients as noncancerous and found that it correctly assigned those patients as negative for cancer with 100% accuracy (16 of 16 cases, 95% CI 79.4–100). Therefore, the high potential for discrimination of AIP from PDAC was validated with an independent confirmatory dataset used in a blinded manner. The serum level of CA19-9 was elevated in 4 (21.1%, 95% CI 7.3−52.4) of the AIP cases in our cohort, while IgG4 levels have been reported to be elevated in 10–30% of PDAC cases [7, 20]. Thus, our proteomic model may be applicable as a novel serological test to discriminate AIP from PDAC in clinical practice. Representative spectra obtained from the AIP and PDAC cases are shown in Figure 4.

### 3.4. Combination of MALDI Proteomic Signature and CA19-9 for Cancer Screening

Our 7-signal proteomic model was able to detect 82.6% (38 of 46, 95% CI 68.6–92.2) of the PDAC patients in the test cohort (Table 2). Moreover, it assigned 78.9% (30 of 38, 95% CI 62.7–90.5) of the patients eligible for an operation to the cancerous group, while 62.5% (5 of 8 and 95% CI 24.5–91.5) of the stage I and II cases were also detected with the identified model. Since it is possible that our 7-signal proteomic model and CA19-9 level are complementary, we investigated whether their combined use would improve the detection rate of patients who may benefit from surgery. The overall sensitivity of CA19-9 (cutoff value, 37 units/mL) alone for stage 0–IVa patients was 71.1% (27 of 38, 95% CI 54.1–84.6), while a combination of our 7-signal proteomic model and CA19-9 level detected 89.5% (34 of 38, 95% CI 75.2–97.1) of operable cases. Notably, for detection of stage I and II PDAC patients, CA19-9 assigned only 50.0% (4 of 8, 95% CI 15.7–84.3) of the cases to the positive group and no additional discrimination power of that marker was observed when combined with our proteomic model. Accordingly, we consider that our 7-signal proteomic model might be more sensitive for detection of early stage PDAC patients than CA19-9, which would improve clinical outcomes following surgical treatment.

### 3.5. Identification of Individual Proteins in the Proteomic Signature

As an initial step toward elucidating the biologic mechanism of the association between the proteomic signature and carcinogenesis, we identified a couple of proteins that correspond to the mass spectrometry signals in the proteomic signature obtained from serum. Extracts from two serum samples of healthy individual were fractionated by reverse phase-HPLC and analyzed by MALDI MS to identify the HPLC fractions that contained proteins corresponding to peaks in the proteomic signature. These selected fractions were subjected to sequence analysis of tryptic peptides by use of MALDI MS. Accordingly, we identified the following proteins as part of the proteomic signature: apolipoprotein A-I  ([M + H]^+^ = 17,250.8 m/z) and C-III ([M+H]^+^ = 8765.1), and transthyretin ([M + H]^+^ = 13761.5).

## 4. Discussion

In the present study, we analyzed the protein expression profiles of plasma specimens obtained from patients with PDAC, as well as acute and chronic pancreatitis cases, and autoimmune pancreatitis (AIP) patients with MALDI MS. Using bioinformatic analysis, we derived 7 MS signals that allowed us to produce a proteomic model for discrimination of PDAC from noncancerous individuals. When we used our proteomic model with both independent test cohort and confirmation group, 62.5% (5 of 8, 95% CI 24.5–91.5) of stage 0–II cases were correctly assigned to the cancerous group, while all AIP patients (19 of 19, 95% CI 82.4–100) were correctly assigned to the noncancerous group. Discrimination of AIP from cancer is obviously important; however it is currently problematic in clinical practice. Although previous reports have shown discrimination power of proteomic signature between PDAC patients and control subjects [21–24], to the best of our knowledge, the present 7-signal proteomic model is the first system of proteomic prediction based upon mass spectrometry found capable to both detect early-stage PDAC cases and discriminate AIP patients.

Early detection is essential for improving the outcomes of PDAC patients. However, those in stages 0–II are difficult to detect with current diagnostic approaches, including computerized tomography scanning, positron emission tomography scanning, and tissue-based diagnostic tests. CA19-9 is a tumor marker widely used for evaluations of therapeutic effects and detection of PDAC recurrence, though it is not considered to be applicable for mass screening when used alone [4, 6, 25, 26]. Recent advances in molecular biology have also revealed that clinical features cannot be adequately characterized or predicted by a single marker. Thus, microarray analysis has been employed to simultaneously investigate the expression levels of thousands of genes and identify mRNA patterns associated with various human diseases including PDAC [27–29]. However, mRNA expression does not always indicate which of the corresponding proteins are expressed or provide information regarding their posttranslational regulation. Moreover, blood and body fluids, such as pancreatic juice and urine, do not contain mRNA. Thus, proteome analysis of such specimens is considered to better reflect the underlying clinical characteristics of human diseases as compared to gene expression profiling, while proteomic technologies including MS have been employed to analyze proteomes in clinical specimens [10–14, 30–32]. Previous proteomics studies of PDAC with healthy controls have shown promising results in distinguishing PDAC, with a sensitivity ranging from 78 to 91% and specificity from 75 to 100% [21–24, 33, 34]. These discrimination power results are better than those obtained with the current CA19-9 marker, while improved diagnostic performance has been observed when serum MS markers were combined with CA19-9 [21, 22, 24]. In the present study, we found that the combination of our 7-signal proteomic model and CA19-9 level improved the positive rate of detection of PDAC patients eligible for surgical resection to 89.5% (34 of 38, 95% CI 75.2–97.1). It is noteworthy that detection of stage I-II cases was also attainable at a sensitivity of 62.5% (5 of 8, 95% CI 24.591.5) without further improvement by adding CA19-9. These results support the usefulness of our 7-signal proteomic model for detection of early stage cases. Since we constructed the present 7-signal model independent from CA19-9, further optimization of selection of a proteomic signature with focus on early detection possibly along with adjustment of the CA19-9 cutoff value is warranted to obtain increased sensitivity. The present 7-signal proteomic model showed high potential to assign inflammatory pancreatic disease patients to the noncancerous group (93.8%; 30 of 32, 95% CI 79.2–99.2). Interestingly, 2 of the misclassified patients suffered from acute pancreatitis; however, all of the patients of chronic pancreatitis and AIP (11 of 11, 95% CI 71.5−100; and 19 of 19, 95% CI 82.4–100) were correctly assigned to the noncancerous group by our proteomic model. Discrimination of AIP from PDAC is difficult in clinical practice, as symptoms such as obstructive jaundice or space occupying lesions in the pancreas are commonly observed in both cases. Actually, most of the AIP patients in this study showed at least one of these symptoms. Our proteomic model distinguished between AIP patients and those with PDAC with high accuracy; thus it is considered to be effective in future clinical applications, especially for selecting those who are eligible for invasive diagnostic procedures followed by inevitably invasive surgical treatment for PDAC. During the course of our study, Frulloni et al. reported that autoantigens against the plasminogen binding protein of helicobacter pylori and ubiquitin-protein ligase E3 component n-recognin 2 were detected in most of the AIP patients tested, as well as a small number of PDAC cases [35]. It would be interesting to combine our proteomic model with testing for those autoantigens for diagnosis of chronic pancreatic diseases.

In this study, 2 acute pancreatitis patients and 14 healthy subjects were assigned to the cancerous group by our 7-signal proteomic model in the training (7 healthy subjects) and test (2 acute pancreatitis patients and 7 healthy subjects) cohorts. Since that time, we have carefully followed their clinical courses of these healthy subjects and found that 5 suffered from cancerous disease within 3 years, including 2 with rectal cancer, 1 with prostate cancer, 1 with hepatocellular carcinoma, and 1 with a metastatic bone tumor from an unknown primary site. In addition, another false positive healthy subject later developed polyposis in the colon. These observations suggest potential relation of our proteomic model with these malignancies, although further in-depth investigations are apparently required to draw definitive conclusions.

Mass spectrometry profiles obtained from complex protein mixtures can contain thousands of data points derived from real protein signatures. However, they can also be contaminated by electronic and chemical noise, variability in instrumentation, and variable crystallization of the matrix, necessitating careful analytical techniques [11, 13, 14]. In the present study, we employed multiple statistical methods and leave-one-out cross-validation to combine differentially expressed proteins with the clinical variables and found that a minimal set of 7 low-molecular weight proteins was sufficient to distinguish between healthy subjects and PDAC patients. The discriminating power of the extracted proteomic signature was further validated using independent test datasets obtained from plasma specimens collected at 2 different institutions. With this protocol, we carefully eliminated accidental identification of overly optimistic and nonbiological/mathematical multivariate signatures within a closed cohort by overfitting.

The primary goal of this study was development of a bioassay applicable to clinical practice for detection of PDAC and discrimination from AIP, as attempts to identify proteins that comprise a proteomic model have not been fully successful to date. However, the high reproducibility of MALDI MS indicates that direct application of its findings would be successful. In the previous study, Koomen et al. reported that a set of 4 peaks could be used to detect PDAC, of which one MS signal was downregulated in PDAC patients and found to be derived from apolipoprotein A-I [23], while Yan et al. found that transthyretin levels were independently associated with PDAC likelihood when obstructive jaundice was considered [36]. Accordingly, our identification of apolipoprotein A-I and transthyretin, which is a constituent of our proteomic model and downregulated in PDAC patients in this study, is in accord with previous reports from different institutes. We also identified the downregulation of apolipoprotein C-III in serum samples obtained from PDAC patients [37, 38]. Further investigations are warranted to identify discriminating proteins for ascertainment of their functional significance. Notably, 2 downregulated peaks (8765 and 13762 m/z), which were previously extracted as proteomic serum markers for lung cancer [39], were also identified as downregulated proteomic signals in PDAC patients in the present study.

Prospective multi-institutional studies with a larger number of patients including those with early-stage PDAC, AIP, and other pancreatic diseases are apparently warranted to validate further significance of our 7-signal proteomic signature for clinical application. Given that it has potential for early detection of PDAC as well as accurate discrimination of AIP, our 7-signal proteomic model may ultimately lead to a reduction in the large number of deaths caused by devastating cancer and also provide better management for chronic inflammatory disease of pancreas.

## Supplementary Material

Supplementary information decribe detailed information about study population and their clinical characteristics, and methods of statistical and proteomic analyses.

## Figures and Tables

**Table 1:** Discrimination of samples in the training cohort according to 7-signal proteomic model.

	Number of cases analyzed	Number of correctly assigned cases (%)	95% C.I.* (%)
All samples	160	134 (83.8)	77.1–89.1
Pancreatic ductal adenocarcinoma	80	61 (76.3)	65.4–85.1
Healthy subjects	80	73 (91.3)	82.8–96.4
age			
≤60	43	30 (69.8)	53.9–82.8
>60	37	31 (83.8)	68.0–93.8
Clinical stage of pancreatic ductal adenocarcinoma patients			
0/I	3	3 (100)	29.2–100
II	8	5 (62.5)	24.5–91.5
III	8	8 (100)	63.1–100
IVa	14	10 (71.4)	41.9–91.6
IVb	47	35 (74.5)	59.7–86.1

*95% confidence interval.

**Table 2:** Discrimination of samples in the test cohort according to 7-signal proteomic model.

	Number of cases analyzed	Number of correctly assigned cases (%)	95% C.I.* (%)
All samples	129	112 (86.8)	79.7–92.1
Healthy subjects	67	60 (89.6)	79.7–95.7
Pancreatic ductal adenocarcinoma (ACCH)	16	13 (81.3)	54.4–96.0
Pancreatic ductal adenocarcinoma (NUH)	30	25 (83.3)	65.3–94.4
Acute pancreatitis (NUH)	2	0 (0)	0–84.2
Chronic pancreatitis (NUH)	11	11 (100)	71.5–100
Autoimmune pancreatitis (NUH)	3	3 (100)	29.2–100
Clinical stage of pancreatic ductal adenocarcinoma patients at ACCH			
0/I	0	NA	NA
II	1	0 (0)	0–97.5
III	3	3 (100)	29.2–100
IVa	4	2 (50)	6.8–93.2
IVb	8	8 (100)	63.1–100
Clinical stage of pancreatic ductal adenocarcinoma patients at NUH			
0/I	1	0 (0)	0–97.5
II	6	5 (83.3)	35.9–99.6
III	13	11 (84.6)	54.6–98.1
IVa	10	9 (90)	55.5–99.7
IVb	0	NA	NA

*95% confidence interval

NA: not available.
