# Supplementary material for: Seven-Signal Proteomic Signature for Detection of Operable Pancreatic Ductal Adenocarcinoma and Their Discrimination from Autoimmune Pancreatitis
Source: Int J Proteomics. 2012 May 14;2012:510397. doi: 10.1155/2012/510397 (PMC3361197; doi:10.1155/2012/510397)
Supplement: Supplementary file 1 — Supplementary information decribe detailed information about study population and their clinical characteristics, and methods of statistical and proteomic analyses. [file 510397.f1.doc]

Seven-Signal Proteomic Signature for Detection of Operable Pancreatic Ductal　Adenocarcinoma and Their Discrimination from Autoimmune Pancreatitis

**Yanagisawa K. et al.**

**Supplementary information**

**SUBJECTS AND METHODS**

**Study Population**

Description of protocol used for recruitment of controls (training and test cohorts) and patients (training and test cohorts) at Aichi Cancer Center, Nagoya, Japan.

The present subjects accrued between January 2001 and November 2005 were selected from those who enrolled in the second version of the Hospital-based Epidemiologic Research Program at Aichi Cancer Center (HERPACC-II), which aimed to enlist all outpatients aged 20 to 79 years old who made their first visit. Details of the study design and subject characteristics have been described elsewhere.[1] In the HERPACC-II, information regarding lifestyle factors was surveyed and a 7-ml blood sample obtained from all first-visit outpatients at Aichi Cancer Center Hospital (ACCH), including both those with and without cancer. Prior to the first examination at our hospital, the patients were questioned regarding their lifestyle when healthy or before the current symptoms developed. Subjects in HERPACC were confirmed the feasibility of using non-cancer outpatients in HERPACC as controls in epidemiological studies on the basis that their general lifestyles were accordant with those of a general population randomly selected from the electoral roll in Nagoya, Aichi Prefecture.[2] The medical background of controls could be another potential source of bias; however, our previous study, which focused on a female population, demonstrated only a limited impact of this variable[3] and little difference would be expected for men. Among the 14 482 blood samples obtained, we identified 96 pancreas cases available for analysis. Eighty of those (55 males, 25 females) were selected for the training cohort and the remaining 16 cases (14 males, 2 females) were used for the test cohort. From the pool of non-cancer subjects among the total cohort of 14 482, we randomly selected 147 as controls, 80 (34 males, 46 females) of whom were assigned to the training cohort and 67 (male: 31 males and 36 females) of whom were assigned to the test cohort.

Description of recruitment of pancreatic ductal adenocarcinoma (PDAC, test cohort) and non-cancerous pancreatic (test and confirmation group) cases at Nagoya University Hospital, Nagoya, Japan.

Plasma samples were obtained from 30 consecutive PDAC patients who had undergone surgical resection with curative intent from May 2004 to July 2006 from the Department of Surgery, Nagoya University Hospital, Nagoya, Japan. All of those samples were assigned to the test set. In addition, plasma samples from 2 acute pancreatitis, 11 chronic pancreatitis, and 19 autoimmune pancreatitis patients who had received medication treatments from September 2003 to August 2009 were obtained from the Department of Gastroenterology, Nagoya University Hospital, Nagoya, Japan, on the basis of availability. All samples from acute and chronic pancreatitis were assigned to the test set, which also contained 3 of the samples from the autoimmune pancreatitis patients. Additional 16 samples were used for further confirmation with special emphasis on autoimmune pancreatitis.

**Proteomic analysis**

Five microliters of non-pre-treated plasma was mixed with 5-nL drops of an energy absorbing matrix solution [saturated Sinapinic acid in water/acetonitrile/trifluoroacetic acid (500:500:1, by volume), which allows molecules to be protonated and desorbed from tissue surfaces). Then, 1-µl mixtures were deposited into individual wells of MALDI MS sample plates (PE Biosystems, Foster City, CA) and dried at room temperature for 5 minutes. Six spots were generated for each plasma-matrix mixture sample and spectra were acquired from all 6 using a 4800 Instrument (Applied Biosystems, Foster City, CA), essentially as described previously.[4, 5] With this analysis, signals in the range of 3000-20,000 mass to charge ratio (*m*/*z*) are considered to identify low molecular weight markers, which are difficult to analyze by two-dimensional gel electrophoresis technology. Each spectrum underwent smoothening to reduce electrical noise and internal calibration with the peaks (molecular weight + one proton [M+H]^+^ = 6631.6 and 13881.0) was performed using Data Explorer software version 4.5 (Applied Biosystems).[4] Additional processes for peak detection and alignment to compare spectra obtained from different patients were conducted using MarkerView software version 1.0 (Applied Biosystems). Custom software was produced to calculate the average signal intensity for each peak across 6 spectra data obtained from each subject. Spectra were normalized by summing the averaged signal intensities across all peaks of each sample and choosing the largest sum from all samples as a global maximum. The sum of total signal intensities of each spectrum was divided by the global maximum to obtain the normalization factor, which was then used to multiply each data point for each spectrum.[4, 5] Previous studies have demonstrated the accuracy and reproducibility of MALDI MS for analysis of human materials (e.g., tumor tissues and serum).[4-9]

**Statistical Methods**

Three distinct statistical methods, Fisher’s exact test, the Kruskal-Wallis test, and a significance analysis of microarray (SAM) test[10], were used to select informative signals for discriminating plasma specimens of PDAC patients from those of healthy controls in the training cohort of 160 individuals. Fisher’s exact test was used to detect informative mass spectrometry signals that were significantly different between low- and high-risk patient groups, when each peak was treated as either present or absent. The total numbers of present and absent calls for mass spectrometry peaks in each subgroup were determined, and mass spectrometry peaks were selected as informative when nonrandom associations were found. The Kruskal-Wallis test was the other nonparametric statistical method used to detect informative mass spectrometry signals that were significantly different between plasma specimens from the PDAC patients and those from the healthy controls. All values for the mass spectrometry peaks were ranked from low to high after disregarding the group to which each value belonged. The smallest number was assigned the rank of 1 and the largest number the rank of *N*, where *N* was the total number of values in all the groups. The rank values in each group were then summed and mass spectrometry peaks of interest were identified as those with statistically significant differences in the sums of the ranks between the subgroups. These two statistical tests were executed with the R statistical programming language.[11, 12] In addition, SAM, a widely used analytic method based on the *t* statistic with permutations, was also used to test each peak value as continuous data using the SAM program version 3.08 developed by Tibshirani et al.[13] The cutoff points were a *P* value corrected with Bonferroni’s correction of less than 0.05 in Fisher’s exact test, a *P* value corrected with Bonferroni’s correction of less than 0.05 in the Kruskal-Wallis test, and a false discovery rate of less than 0.1% in the SAM test. Mass spectrometry signals that met at least one of the three selection criteria were further analyzed.

To construct a generally applicable proteomic classifier without specifically over-fitting it to the training cohort, we used a weighted voting algorithm, a well-established technique for supervised classification in which each weight value is calculated as the signal-to-noise ratio and leave-one-out cross-validation strategy.[14] The average number of misclassified patients for the *N*-signal model was calculated as follows. The training set was divided into 1 subject and the other subjects. Then, the weight (signal-to-noise metric) of each mass spectrometry signal was calculated using data from the majority subsets, while at the same time each mass spectrometry signal was ranked on the basis of the absolute value of the SAM. The top *N*-ranked mass spectrometry signals by rank were used to construct a prognosis classifier. An *N*-signal model was used for the 1 subject and the number of misclassified patients ($N_{error N,P,M}$, where *N* is the number of signals used for the classifier, *P* is the *P*th permutation, and *M* is the *M*th subset) was calculated. This process was repeated for each subject individually and the cross-validated learning error was calculated as the sum of the number of misclassified patients in each process.

The proteomic prediction model was assessed as follows. When the plasma specimens from PDAC patients and healthy controls were defined as class 0 and 1, respectively, the signal-to-noise statistic (*Sx*; *x* = gene *x*) was calculated as *Sx* = (μ*_class0_* – μ*_class1_*/*σ_class0_* + *σ_class1_*), where μ*_class0_* is the mean value and *σ_class0_* the standard deviation for that profile in all samples in class 0. We selected the top 7 ranked mass spectrometry profiles on the basis of the absolute values for *Sx* of each gene. A weighted-voting classification algorithm was used to predict outcome with data from the mass spectrometry signals selected as described above, then the resulting outcome classifiers were tested using leave-one-out cross-validation. With this scheme, the algorithm can also be used to find the decision boundaries between the class means as *b_x_* = (μ*_class0_* + μ*_class1_*)/2 for each gene, in addition to computing *Sx*. To predict the class of a test sample γ, each profile *x* in the predictive mass spectrometry profile set has a vote (*V_x_*) that is based on the expression in this sample (*g_x_*) and *b_x_* [i.e., *V_x_* = *S_x_*(*g_x_* – *b_x_*)], and the final vote for class 0 or 1 is sign (Σ*_x_V_x_*).

It is possible that unintended biased re-substitution or partial cross-validation can result in underestimation of the error rate after cross-validation, thus the performance of any class prediction rule is best assessed by applying the rule created by use of 1 dataset (the training set) to an independent dataset (the validation or test set).[15] The proteomic classifier constructed with the training dataset of 160 individuals was then validated using a completely independent validation of 145 individuals.

The agglomerative hierarchical clustering algorithm was applied to investigate the pattern among the statistically significant discriminator proteins as well as the biological status with Eisen’s software.[16]

**Supplementary Table S1. Clinicopathologic characteristics of patients with PDAC and non-cancerous pancreatic disease from Aichi Cancer Center and Nagoya University Hospital in the training and test cohort**

| Variable | No. of cancer pts in training set (ACC) | No. of cancer pts in test set (ACC) | No. of cancer pts in test set  (NUH) | No. of pts with non-cancerous diseases in test set  (NUH) | No. of healthy subjects in training set  (ACC) | No. of healthy subjects in test set  (ACC) | |
| --- | --- | --- | --- | --- | --- | --- | --- |
| All patients | 80 | 16 | 30 | 16 | 80 | 67 | |
| Age (y.o) | | | | | | | |
| ≤ 60 | 43 | 8 | 2 | 8 | 53 | 34 | |
| > 60 | 37 | 8 | 28 | 8 | 27 | 33 | |
| Sex | | | | | | | |
| Male | 55 | 14 | 19 | 12 | 34 | 31 | |
| Female | 25 | 2 | 11 | 4 | 46 | 36 | |
| CA19-9 (units/ml) | | | | | | | |
| ≤ 37 | 11 | 5 | 9 | 13* | NA | NA | |
| > 37 | 69 | 11 | 21 | 1* |  |  |  |
| pT^#^ status | | | | | | | |
| Tis/1 | 3 | 0 | 1 | NA | NA | NA | |
| T2 | 0 | 1 | 0 |  |  |  |  |
| T3 | 40 | 8 | 19 |  |  |  |  |
| T4 | 32 | 6 | 10 |  |  |  |  |
| Tx§ | 5 | 1 | 0 |  |  |  |  |
| pN^#^ status | | | | | | | |
| N0 | 24 | 5 | 14 | NA | NA | | NA |
| N1 | 43 | 6 | 16 |  |  |  |  |
| Nx§ | 13 | 5 | 0 |  |  |  |  |
| pStage^#^ status | | | | | | | |
| 0 / I | 3 | 0 | 1 | NA | NA | | NA |
| II | 8 | 1 | 6 |  |  |  |  |
| III | 8 | 3 | 13 |  |  |  |  |
| IVa | 14 | 4 | 10 |  |  |  |  |
| IVb | 47 | 8 | 0 |  |  |  |  |
| Non-cancerous pancreatic diseases | | | | | | | |
| Acute pancreatitis | NA | | | 2 | NA | | NA |
| Chronic  pancreatitis |  |  |  | 11 |  |  |  |
| Autoimmune  pancreatitis |  |  |  | 3 |  |  |  |

^#^pT = pathologic primary tumor; pN = pathologic lymph node status; pStage = pathologic disease stage.

§When the M status met 1 or T status met 4, further evaluation of T or N status was not carried out in some cases.

*Data from 2 cases were not available.

NA: not available

**Supplementary Table S2. Clinicopathologic characteristics of autoimmune pancreatitis patients in validation and confirmatory cohorts**

| Variable | No. of patients in test cohort (NUH) | No. of patients in confirmation group (NUH) |
| --- | --- | --- |
| All patients | 3 | 16 |
| Age (y.o) | | |
| ≤ 60 | 1 | 7 |
| > 60 | 2 | 9 |
| Sex | | |
| Male | 3 | 16 |
| Female | 0 | 0 |
| CA19-9 (units/ml) | | |
| ≤ 37 | 3 | 12 |
| > 37 | 0 | 3 |
| NA | 0 | 1 |
| IgG4 (mg/dl) | | |
| < 135 | 0 | 0 |
| ≥ 135 | 3 | 16 |

NA: not available

**References**
